# A possible case of spontaneous *Loa loa *encephalopathy associated with a glomerulopathy

**DOI:** 10.1186/1475-2883-5-6

**Published:** 2006-05-10

**Authors:** Tuna Lukiana, Madone Mandina, Nanituma H Situakibanza, Marcel M Mbula, Bompeka F Lepira, Wobin T Odio, Joseph Kamgno, Michel Boussinesq

**Affiliations:** 1 Département de Médecine Interne, Cliniques Universitaires de Kinshasa, BP 123, Kinshasa IX, République démocratique du Congo; 2 Centre Pasteur du Cameroun, BP 1274, Yaoundé, Cameroun; 3 Institut de Recherche pour le Développement, Département Sociétés et Santé, 75480 Paris Cedex 10, France

## Abstract

It is well known that renal and neurological complications may occur after antifilarial treatment of patients infected with *Loa loa*. Conversely, spontaneous cases of visceral complications of loiasis have been rarely reported. A 31-year-old Congolese male patient who had not received any antifilarial drug developed oedema of the lower limbs, and then transient swellings of upper limbs. Two months after, he developed troubles of consciousness within several hours. At hospital, the patient was comatose with mild signs of localization. Laboratory tests and an abdominal echography revealed a chronic renal failure due to a glomerulopathy. Three weeks after admission, *Loa *microfilariae were found in the cerebrospinal fluid, and a calibrated blood smear revealed a *Loa *microfilaraemia of 74,200 microfilariae per ml. The level of consciousness of the patient improved spontaneously, without any specific treatment, but several days after becoming completely lucid, the patient died suddenly, from an undetermined cause. Unfortunately, no biopsy or autopsy could be performed. The role of *Loa loa *in the development of the renal and neurological troubles of this patient is questionable. But the fact that such troubles, which are known complications of *Loa *infection, were found concomitantly in a person harbouring a very high microfilarial load suggests that they might have been caused by the filarial parasite. In areas endemic for loiasis, examinations for a *Loa *infection should be systematically performed in patients presenting an encephalopathy or a glomerulopathy.

## Findings

If the classical signs of loiasis (itching, eye worm and Calabar swellings) are well known [1,2], the more serious manifestations of the infection, i.e. the visceral ones, are still poorly documented. The renal or the neurological complications which have been reported by various authors, often occurred after an antifilarial treatment with diethylcarbamazine (DEC) or ivermectin [3-6]. In contrast, the descriptions of spontaneous complications are fairly rare and, in such cases, their relationship with the infection by *Loa loa*, and/or their spontaneous nature are sometimes questionable.

Various neurological manifestations, without any associated trouble of consciousness, have been attributed to loiasis: headaches, asthenia, somnolence, motor deficits, troubles of sensation, and cerebellar, vestibular, or psychiatric disorders [7]. But, to our knowledge, the only cases in which real troubles of consciousness occurred in patients infected with *Loa loa *and who had possibly, probably or certainly not received any filaricidal treatment are those reported by Bonnet, [8], Kivits, [9], Gallais *et al*., [10] and then Carayon *et al*., [11], Cauchie *et al*., [12], Same-Ekobo *et al*., [13], and Negesse *et al*., [14]. The patient described by Bonnet, [8] had certainly not taken DEC or ivermectin because the first use of these antifilarial drugs in humans took place in 1947 and 1980, respectively. In the cases described by Cauchie *et al*., [12] and Same-Ekobo *et al*., [13], a history of treatment before the appearance of the troubles is fairly unlikely, because a careful interview of the relatives had been performed. The situation is probably the same for the patient described by Gallais *et al*., [10], who was a Chadian soldier posted in Indochina. Conversely, it is quite possible that one or several of the patients described by Kivits, [9] had taken DEC within the days preceding their hospitalization. Indeed, as noticed by Fain, [15], DEC was already fairly widely available in the Mayumbe area (Bas-Congo province, Democratic Republic of the Congo, DRC) as soon as 1949–1950 and, as the neurological complications of DEC were not known at that time, the author has naturally not asked the question to the relatives of whether the patients had received the drug or not. Besides this, the patient described by Negesse *et al*., [14] had taken a "native medicine", and the authors raised the hypothesis that the latter might have contained DEC. Thus, it appears that the spontaneous cases of *Loa loa *encephalopathy are exceptional.

Concerning the renal manifestations, long ago a number of authors have reported the presence of an albuminuria, and sometimes of a microscopic haematuria, in some patients infected with *Loa loa *[16,17]. In several cases, a renal biopsy had been performed before the administration of the antifilarial treatment, and the lesions observed corresponded to glomerulopathies of various types [18-22]. However, here again, the detailed observations are fairly rare.

In this context, it seemed interesting to us to present in detail the following case of a patient who was heavily infected with *Loa loa *and who presented, successively, a glomerulopathy and an encephalopathy.

Mr BL, 31 years old, was a Congolese (DRC) male who was born in Kinshasa and who lived since 1998 in Muanda (Bas-Congo Province). On the 11^th ^of October, 2004, he was brought by his family to the General provincial Hospital of Kinshasa because he showed troubles of consciousness that had started on the previous day. Before mid-2004, the patient had no special medical history, except haemorrhoids. In July 2004, he complained of abdominal pains, which were more important in the pelvic region, and accompanied by diarrhoea. He treated himself with the traditional plants he took usually to treat his haemorrhoids. This was followed by an improvement in his diarrhoea but some time later the patient noticed the appearance of oedema of both lower limbs and he complained of a slight distending of abdomen. He treated the oedemas by local applications of a paste made of a mixture of plants. The oedemas diminished a little, but the patient then noticed the appearance of transient swellings of both upper limbs, accompanied with itching, that he treated with promethazine oral syrup during one week. The itching disappeared but the persistence of the oedema of the lower limbs one month after their appearance prompted the patient to go to Kinshasa so as to receive an appropriate treatment. On the 20^th ^of September, he went to a health centre and, in addition to the oedema, a fever was noticed by the nurse. A blood smear was prepared, which showed a light infection with *Plasmodium *(noted as + on the laboratory register), and a treatment with oral quinine and paracetamol was given. By the 8^th ^of October, the patient showed a fever in the evening during two days. This was then accompanied with an oliguria, the appearance of an oedema of the face on waking, and an asthenia. In the morning of the 11^th^, the patient presented a functional impairment and troubles of consciousness which prompted his family to bring him to the General provincial Hospital of Kinshasa (GHK).

At admission to the GHK, on the 11^th ^of October (Hospital Day 1, HD1), the patient presented with confusion, a swelling of the face, and a pitting oedema of the lower limbs. The temperature was not measured but the patient had a mild fever to the touch. The abdomen was soft, and no organomegaly was found. The blood pressure was 110/80 mmHg and the respiratory frequency, 20 breaths/min. There was no neck stiffness, the palpebral conjunctivae were normal, and bronchial breath sounds were found at auscultation. The rest of the examination was normal. The results of the laboratory tests performed the same day were as follows: low haematocrit, at 28% (normal value: 42–54%); leukocytosis at 12,000/mm^3 ^(normal: 4000–10,000/mm^3^); normal glycemia at 0.98 g/l; presence of *Plasmodia *in the thick blood smear (1 to 10 parasites per microscope field); albuminuria +++ on dipstick urine analysis; absence of glycosuria; and presence, in the urine, of red blood cells (10/field) and of leukocytes (10/field; normal value:< 5/field). A diagnosis of nephrotic syndrome associated with a severe malaria was proposed. An intravenous treatment with furosemide, cefotaxime, and quinine, was started. A permanent urine catheter was put in place, in order to measure diuresis. Additional biological examinations, and an abdominal echography were prescribed.

On HD2 and HD3, the patient was in stage I coma, i.e. responded to pain by more or less comprehensible words. After an episode of agitation during the night of the HD2, he was calm, and still subfebrile to the touch. The rest of the condition was similar to the one recorded at admission. On HD3, the sedimentation rate was high, at 120 mm per hour; the leukocyte count had become normal at 4800/mm^3^; and a new blood smear still showed the presence of *Plasmodium*, but at a very low density (1 parasite per 100 fields). On the HD4, an echography was performed, whose results were as follows: "kidneys of normal size and morphology, but with a marked loss of the cortico-medullar differentiation, making them almost white; liver increased in volume, but normal in its morphology and homogeneous in its structure; presence of an ascites, principally pelvic". It was concluded that the images were strongly suggestive of a bilateral glomerulopathy, associated with an hepatomegaly which was probably reactional to the renal disease. On HD5, the patient, whose clinical condition was stable, was transfered to the *Cliniques Universitaires de Kinshasa *(CUK) for more extensive investigations.

On admission to the Emergency Unit of the CUK, the patient was in stage II coma (i.e. he responded to pain by no or incomprehensible sounds, but by an appropriate motor response), without neck stiffness. He still presented a swelling of the face, with an important oedema of the eyelids which obstructed completely his left eye. The oedema of the lower limbs was still present, the abdomen was slightly distended, and a moderate ascites was found. The temperature was 37.7°C, the blood pressure was 130/90 mmHg, and the respiratory frequency was 20/min. The results of the blood tests done on HD5 were as follows: marked anemia, with an haemoglobin concentration of 7.5 g/100 ml (normalvalues: 13–17); haematocrit still lower than at HD1, at 22%; normal leukocyte count, at 6800/mm^3^, with 79% neutrophils, 18% lymphocytes, and 3% eosinophils; urea: 1.05 g/l(normal: 0.10–0.42); creatinine: 29 mg/l(normal: 5–20); creatinine clearance: 27.6 ml/min(normal: > 60 ml/mn); glycemia: 1.13 g/l; sodium concentration: 138 mEq/l; potassium: 4.6 mEq/l. These results showed that the patient presented a marked chronic renal failure, with albuminuria and haematuria which, together with the echographic findings, suggested that it was due to a glomerulopathy. The hypothesis is raised that the renal failure might have been provoked by a possible nephrotoxicity of the traditional plants taken by the patient. Besides this, owing to the biological results, the degree of the troubles of consciousness and the clinical condition in general did not suggest an uraemic coma. The treatment implemented at the GHK, which included intravenous furosemide, cefotaxime and quinine was continued. A renal biopsy was prescribed, but later was not performed because of financial considerations. On HD6, the hypothesis was raised, that the neurological troubles would be due to a bacterial encephalitis or to a severe malaria. A new blood smear was prepared, in which no *Plasmodium *was found. On HD7, the calcemia was measured; it was at 4.6 mEq/l, i.e. close to the lower limit of normal (4.5–5.5); the bicarbonates were at 24 mmol/l (normal: 18 to 33). On HD8, a complete clinical examination was done again. Besides the troubles of consciousness, the oedema of the face and of the lower limbs, and the absence of renal bruit at auscultation, the examination showed several neurological signs which had not been noticed previously: a left facial paralysis, a convergent strabism of the right eye, an hypotonia of the right hemibody, a bilateral Babinski sign, and normal tendon reflexes. The hypothesis was raised that the neurological troubles might have been caused by an expansive intracranial process, including a cerebral abcess possibly related to an HIV infection. The patient was transfered to the Intensive Care Unit of the CUK.

When he was admitted to the unit, on HD8, the patient was still in a stage II coma, without fever nor meningeal signs; the blood pressure was 120/80 mmHg, and the respiratory frequency, 24/min. The liver function tests performed the same day were normal (conjugated bilirubin: 9 mg/l; unconjugated bilirubin: 3 mg/l; ALAT: 3 UI/l; ASAT: 16 UI/l). A lumbar puncture was also performed and the cerebrospinal fluid (CSF) obtained was crystal clear, and showed no evidence of infection either at direct microcopic examination or after culture. A fundus examination showed narrowed retinal arteries, perivenous haemorrhages, several cotton-wool exudates, a slight macular oedema in the left eye, and well coloured and well limited papillae. On HD9, the neurological condition was stable, whereas the oedema of the lower limbs seemed to have regressed a little. New blood tests showed that the sedimentation rate was still high (150 mm per hour), an increase in the haemoglobin concentration (8.9 g/100 ml), a calcemia at 4.2 mEq/l and bicarbonates at 20 mmol/l. The results of the CSF tests were as follows: red blood cells: 79/mm^3^; leukocytes: 2/mm^3^; glucose: 0.68 g/l; proteins: 0.64 g/l (normal: 0.20–0.40); chloride: 126 mmol/l (normal: 120–130 mmol/l). The association of a high concentration of proteins with a low density of leukocytes was characteristic of an albumino-cytologic dissociation. A nasogastric tube was set up, in order to feed the patient with 400–600 ml of gruel and 300–400 ml of water per day, following the diuresis measured daily.

On HD10, the patient's clinical condition was similar with, in addition, a neck stiffness and an abolition of the tendon reflexes. The sedimentation rate was 124 mm per hour. As the possible intracranial process could be due to an abscess caused by pyrogens, the ongoing treatment with cefatoxime and quinine was completed by metronidazole. On HD11, the glycemia was at 1.47 g/l. On HD13, the hypothesis was raised that the neurological troubles might be due to a thrombo-embolic complication of the nephrotic syndrome. A cerebral CT scan was prescribed, but was not performed. Quinine treatment was stopped. On HD15, the patient who had remained apyretic until then, showed a fever at 38.6°C; the temperature became normal again two days after. On HD17, the leukocyte count was 7900/mm^3^, with 70% neutrophils, 20% lymphocytes and 10% eosinophils. On HD18, the treatment with metronidazole was interrupted, and though it was most unlikely that the condition would be related to stroke, a treatment by piracetam and multivitamins was started, whereas cefatoxime was continued. In spite of the continuing nursing care since the start of hospitalization, bedsores on the trochanters appeared on HD19. The patient was still in stage II coma. Bedsores on the coccyx apperared on HD23. On HD24, the urine protein excretion, quantified for the first time, was 1.30 g/24 h (normal: < 150 mg/24 h). On the same day, the blood pressure was found to be elevated on several instances, with diastolic values reaching or exceeding 100 mmHg. On HD25, the Glasgow coma score was 7/15 (eye opening: 4/4; motor response: 1/6; verbal response: 2/5). On the same day, a new lumbar puncture was performed, and the CSF was examined after double centrifugation to detect trypanosomes. No trypanosome was found, but three *Loa loa *microfilariae were counted. The other results concerning the CSF were as follows: glucose: 0.97 g/l; proteins: 0.98 g/l; chloride: 130 mEq/l; red blood cells: 2560/mm^3^; leukocytes: 4/mm^3^; absence of infection at direct examination and after culture; China ink stain to detect *Cryptococcus neoformans *capsules negative. Though the result on the red blood cells might mean that the CSF was contaminated with blood, and thus microfilariae, from a vessel injured during the tap, the presence of these *Loa loa *microfilariae suggested that the renal and neurological conditions of the patient might correspond to complications of a *Loa loa *infection. The same day, an HIV serology was done, which was found negative.

The treatment by piracetam and vitamins was continued, and a treatment by enalapril maleate (Envas) and nadroparine calcium (Fraxiparine) was started to stabilize the blood pressure and prevent thromboembolic complications due to prolonged bed rest, respectively. Two options were considered to treat the *Loa loa *infection: diethylcarbamazine (DEC) associated with corticosteroids, and a treatment with Vofil – a speciality made of medicinal plants, manufactured and marketed in DRC – in association with astemisol, an antihistaminic. These treatments not being available, they were not administered to the patient. On HD27, the patient's clinical condition improved: he was in stage I coma. On HD28, the appearance of a productive cough prompted the medical team to start a treatment with carbocysteine syrup, which was replaced by acetylcysteine on HD30. On HD29, the haematocrit was 23% and the haemoglobin concentration was 8.2 g/100 ml. Treatment with nadroparine calcium was stopped, and replaced with aspirin at low dose. The clinical condition remained stable until HD34, when the patient was found lucid and coherent, to our great surprise. The nasogastric feeding tube was removed. During the following days, the patient was asthenic and appeared to be slightly out of touch with the reality, but he responded to verbal command and his level of consciousness improved progressively. He still showed mild pitting oedema of the lower limbs. A calibrated (50 μl) blood smear was prepared on HD35, and showed a high infection with *Loa loa *microfilariae (74,200 microfilariae per ml of blood). This result, and the favourable evolution of the condition, led to the project of treating the patient with DEC being abandoned. At that time, the patient was treated with piracetam per os, enalapril maleate, aspirin, multivitamins, and nursing care for his bedsores. We considered the possibility, as soon as the patient's condition would be satisfactory, of starting a long-term treatment with albendazole in order to decrease his *Loa *microfilaraemia progressively. On HD39, the patient left the Intensive Care Unit. His state of consciousness permitted questioning him in detail about the development of his troubles. He clearly stated that he was well aware that a mass ivermectin treatment had been organized in some areas of the Bas-Congo province in the first months of 2004, but that for his part he did not take this treatment nor any other antifilarial drug. On HD45, during nursing care for his bedsores, the patient presented convulsions, and died suddenly.

The relationships between the different troubles that the patient has developed, in a sequential manner, within three months between July and October 2004, cannot be fully ascertained. The fact that he presented a very high *Loa loa *microfilarial load constitutes one of the crucial elements of the picture. Owing to the prolonged effect of DEC and ivermectin on the *Loa *microfilaraemia, [23], this high load strongly suggests that the patient, as he informed us when he could be questioned, had probably not received any antifilarial treatment within the last months, or even years. It is also noteworthy that the oedema of the lower limbs had appeared shortly before the appearance of signs that were suggestive of loiasis.

During the first two months, the prevailing sign presented by the patient was the oedema of his lower limbs. The laboratory tests performed within the first days of hospitalization demontrated a significant decrease in the creatinine clearance, and thus showed that the troubles were related to a renal failure. In addition, the echographic findings, and the presence of an important albuminuria associated with a microscopic haematuria led rapidly to a diagnosis of glomerulopathy. As no renal biopsy could be performed, it is difficult to determine the exact type of renal lesions, or their aetiology. However, the clinical and biological examinations that were performed allow us to exclude several aetiologies: diabetic nephropathy, HIV-associated nephropathy, gout nephropathy. Besides this, a number of authors have reported cases of patients who presented, as ours, an infection with *Loa loa *and a renal disease with oedema of the lower limbs [20-22].

In the present case, the troubles of consciousness appeared fairly rapidly, within only several hours, after a period of two days during which the patient had presented an evening fever and headaches. The presence of a *Plasmodium *infection could explain the fever and possibly the neurological symptoms; and this prompted us to start treatment with quinine. However, the absence of improvement during the following days strongly suggests that the troubles were not due to a severe malaria. The fact that the development of the coma was preceded by headaches, and the presence of mild signs of localization led us to consider the possibility of an intracranial expansive process. However, the absence of arterial hypertension, of any signs of intracranial hypertension in the fundus, or of any clinical improvement within the days following implementation of a wide-spectrum antibiotherapy, as well as the "spontaneous" subsequent improvement in his clinical condition, finally led us to consider this possibility as unlikely. The finding of *Loa loa *microfilariae in the CSF, as well as the very high *Loa *microfilaraemia, consitute arguments in favour of a diagnosis of *Loa *encephalopathy. In addition, the haemorrhages and cotton-wool exudates found in the retina of our patient were similar to those reported both in patients who developed a *Loa *encephalopathy after ivermectin treatment [24], and in individuals without history of antifilarial treatment but harbouring a very high *Loa *microfilaraemia [25]. However, one should notice that the CSF contained also a fairly high number of red blood cells, and thus that it could have been contaminated by blood and microfilariae coming from a vessel injured during the puncture.

In the absence of histological results, it is difficult to affirm that the renal and neurological troubles developed by our patient were indeed caused by his high *Loa loa *infection. An improvement after an antifilarial treatment would have constituted a strong argument in favour of this possibility. But due to his very high microfilaraemia, such a treatment would have probably induced, in our patient who was still very weak, a serious adverse reaction that would have led to a new aggravation of the picture. We thus prefered to postpone the antifilarial treatment, which finally could not be administered because of the sudden and expected outcome. At the end, the main argument that makes us think that the renal and neurological troubles might have been caused by *Loa loa *is the fact that they are both known complications of *Loa *infection, and that they occurred concomitantly in a patient harbouring a very high microfilaraemia.

The pathogenic mechanisms associated with the *Loa*-related serious adverse reactions after an antifilarial treatment are imperfectly documented. As far as ivermectin is concerned, it is probable that the drug provokes a fairly rapid paralysis of the microfilariae, and that the latter, floating passively in the blood circulation, may finally provoke embolisms in the capillaries, especially in the brain [24]. In addition, it has been shown that ivermectin provokes a passage of the blood *Loa loa *microfilariae into the CSF and into the urine [4]. These phenomena might explain the neurological and renal complications that occur sometimes after treatment. In contrast, the factors associated with a spontaneous development of a *Loa*-related encephalopathy or glomerulopathy are unknown. Several authors have raised the hypothesis that an intercurrent or a concomitant affection weakening the blood-brain barrier might provoke lesions at the vascular level and/or facilitate the passage of the microfilariae into the brain tissue. This could be the case with trypanosomiasis [8,26], syphilis [8], an acute encephalitis [9], an infection associated with flu symptoms [12,27], or a local suppuration [9]. The possible role of malaria should also be considered. Indeed, when admitted to hospital, our patient was also infected with *Plasmodium*, as was one of the cases reported by Kivits, [9]. Besides this, the child whose history was described by Same Ekobo *et al*., [13] had presented, three days before the appearance of the neurological troubles, "a fever eliminated by quinine treatment". As *Plasmodium *infection is also very frequent in those areas endemic for *Loa loa*, these observations may be purely coincidental. However, the possible role of *Plasmodium *as a cofactor facilitating the development of a post-ivermectin *Loa *encephalopathy has also been raised [6], and a study is ongoing in Cameroon to investigate this point. Lastly, one may imagine that a cranial traumatism could also provoke capillary lesions that would facilitate the passage of the microfilariae into the cerebral parenchyma; this could explain the clinical picture described by Gallais *et al*., [10] in a soldier who "had received a blow with a butt on his head".

In the case we have presented, the most surprising event was probably the rapid improvement in the neurological condition, within only several days. As pointed out above, the interval between the start of quinine treatment and this improvement is too long for malaria being the cause of the condition. On the other hand, the clinical changes we have observed were similar to those described by Cauchie *et al*., [12] in a patient who had developed a *Loa *encephalopathy that was probably spontaneous. In the latter case, "the troubles of consciousness lasted five days, and then disappeared completely, without any specific treatment" (the patient died subsequently, when the diagnosis of loiasis was made, and after several days of treatment with DEC and corticosteroid, that provoked a coma that was, this time, fatal). It seems that, even in the absence of previous antifilarial treatment, *Loa *microfilariae can spontaneously penetrate into the brain tissue, where they may induce a cellular immunological reaction [12,28]. Though difficult to demonstrate, one may consider the possibility that the infiltrates and granulomas which form around the parasites may then be resorbed progressively, and that, even if the lesions are diffuse, this may lead to a clinical improvement. Besides this, the sudden death of our patient, when the medical personnel was providing care for his bedsores, constitutes also an enigma. In the absence of autopsy, we can only propose an hypothesis to explain this. One may consider the possibility of a cerebral embolism, which might have constituted a complication of the nephrotic syndrome, and/or facilitated by the interruption of the anticoagulant treatment. Lastly, such an embolism might also have been facilitated by the very high microfilaraemia.

In conclusion, we have described the case of a patient who presented a high infection with *Loa loa *and who developed, successively, two visceral manifestations which could be caused by this infection. The imputability of *Loa loa *in the clinical condition of our patient is questionable. This is due to the fact that the spontaneous visceral complications of loiasis are little known and that, in consequence, the presence of a *Loa *infection is rarely examined in patients presenting an encephalopathy or a glomerulopathy. In the present case, the diagnosis of loiasis was made only after three weeks of hospitalization. Besides this, once the diagnosis of *Loa *infection had been made, the investigations that would permit us to connect the clinical picture and the filariasis are rarely made. This is sometimes due to the cost of the required examinations but we do think that two of them should be made on patients who are infected with *Loa loa *and who present an encephalopathy: an examination of the fundus, to see whether there are typical retinal haemorrhages, and a direct examination of centrifuged sample of CSF. In patients with *Loa *infection and developing a glomerulopathy, it is desirable, if possible, to perform a renal biopsy.

## Abbreviations

ALAT Alanine Amino-Transferase

ASAT Aspartate Amino-Transferase

CSF Cerebrospinal fluid

CUK Cliniques Universitaires de Kinshasa

DEC Diethylcarbamazine

DRC Democratic Republic of the Congo

GHK General provincial Hospital of Kinshasa

## Competing interests

The author(s) declare that they have no competing interests.

## Authors' contributions

TL, MM, NHS, MMM, BFL and WTO took care of the patient at the CUK, and commented on the manuscript. TL and MM drafted the first version of the case report, and exchanged ideas and information with MB. JK examined the patient, quantified his level of *Loa *infection, and commented on the manuscrit. MB provided information and advice to the medical team on the management of the patient, and wrote the background, the discussion, and the final version of the manuscript.
